# Duodenum inversum: a rare cause of nausea and epigastric pain

**DOI:** 10.1259/bjrcr.20210144

**Published:** 2022-02-02

**Authors:** Chin Harn Yap, Danielle Coupland, John Au, Smita Raju

**Affiliations:** 1 Royal Adelaide Hospital, Port Rd, Adelaide, Australia; 2 The University of Adelaide, School of Medicine, Adelaide, Australia

## Abstract

Duodenum inversum is a rare congenital anomaly of unknown aetiology characterised by the proximal duodenum travelling posteriorly and superiorly prior to crossing midline. Clinical presentations include epigastric pain, nausea, and abdominal distension. It can be associated with duodenitis, acute pancreatitis, peptic ulcer disease and functional biliary obstruction. In this case report, we discuss a 77-year-old male who presented with hematemesis and epigastric pain secondary to duodenitis, for which he had a CT scan of the abdomen which demonstrated duodenum inversum. Despite the rarity of the condition and its common omission from differential diagnoses, the ability to recognise duodenum inversum is important for radiologists, especially considering its implications in clinical management. If not diagnosed correctly, it may result in unnecessary hospital admissions, dietary restrictions, and perhaps even unnecessary surgery. In this case, the radiological diagnosis of duodenum inversum using CT allowed for conservative medical management and prevented surgical intervention.

## Case presentation

A 77-year-old male presented with hematemesis associated with nausea and abdominal pain. He had a history of hypertension, bilateral hip and knee replacements and chronic history of non-steroidal anti-inflammatory and aspirin use. Vital signs upon arrival at the emergency department were within normal limits with a blood pressure of 104/63  mmHg, heart rate of 71 beats per minute, oxygen saturation of 95% while breathing ambient air, temperature of 37°C, and respiratory rate of 16 respirations per minute. Abdominal examination revealed a non-distended abdomen with tenderness in the epigastrium. There was no guarding, rebound tenderness, pulsatile, or palpable mass. Laboratory results were unremarkable with a lipase of 50 [0–60 U l^−1^], bilirubin of 11 [2–24 U l^−1^], alkaline phosphatase of 63 [30–110 U l^−1^], gamma-glutamyl transferase of 23 [0–60 U l^−1^], aspartate aminotransferase of 21 [0–45 U l^−1^] and alanine aminotransferase of 10 [0–55 U l^−1^].

## Investigations

**Figure 1.**
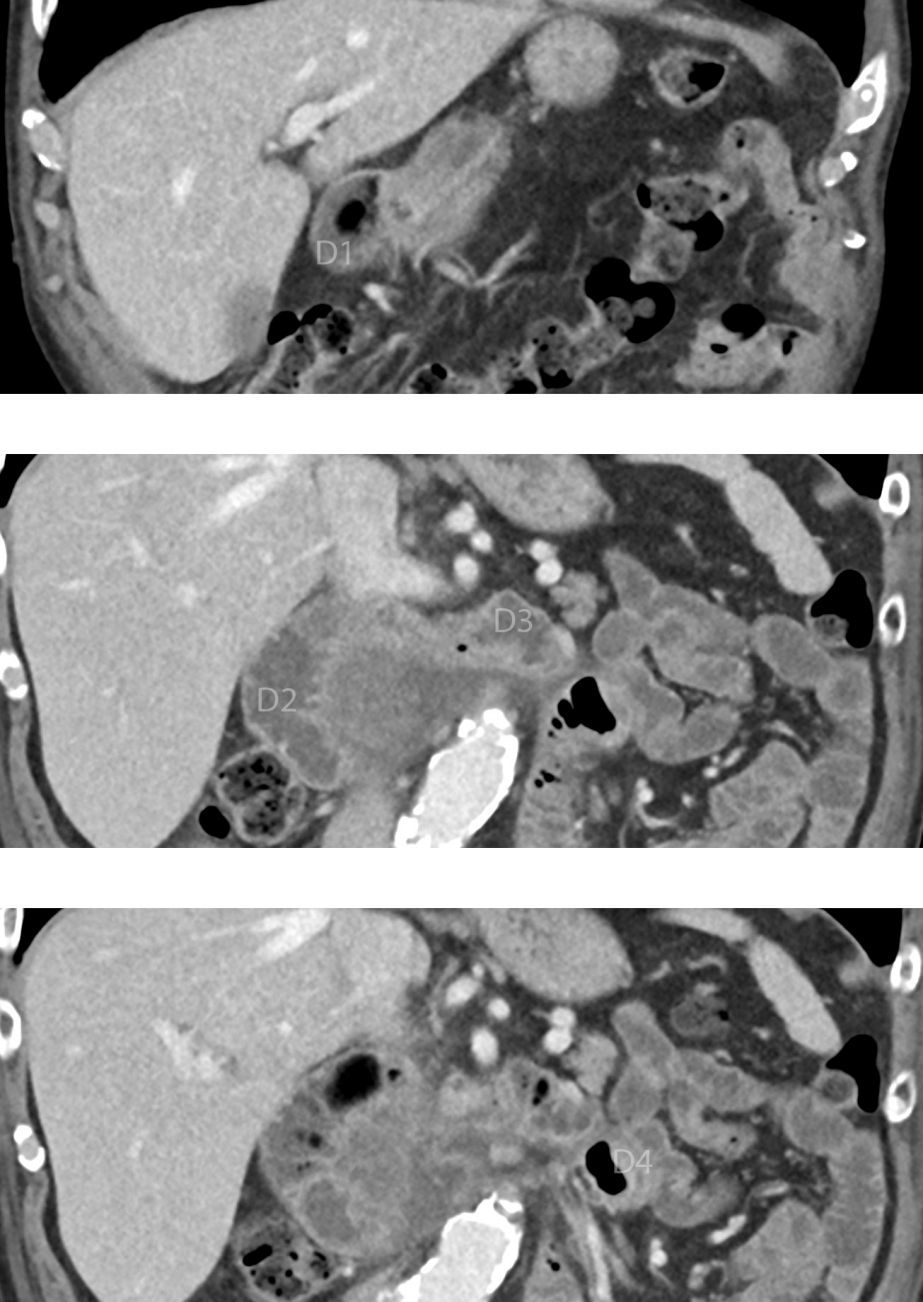
Serial CT coronal images demonstrating the D3 segment of the duodenum crossing midline in a high position in relation to D1 and the DJ flexure.

**Figure 2.**
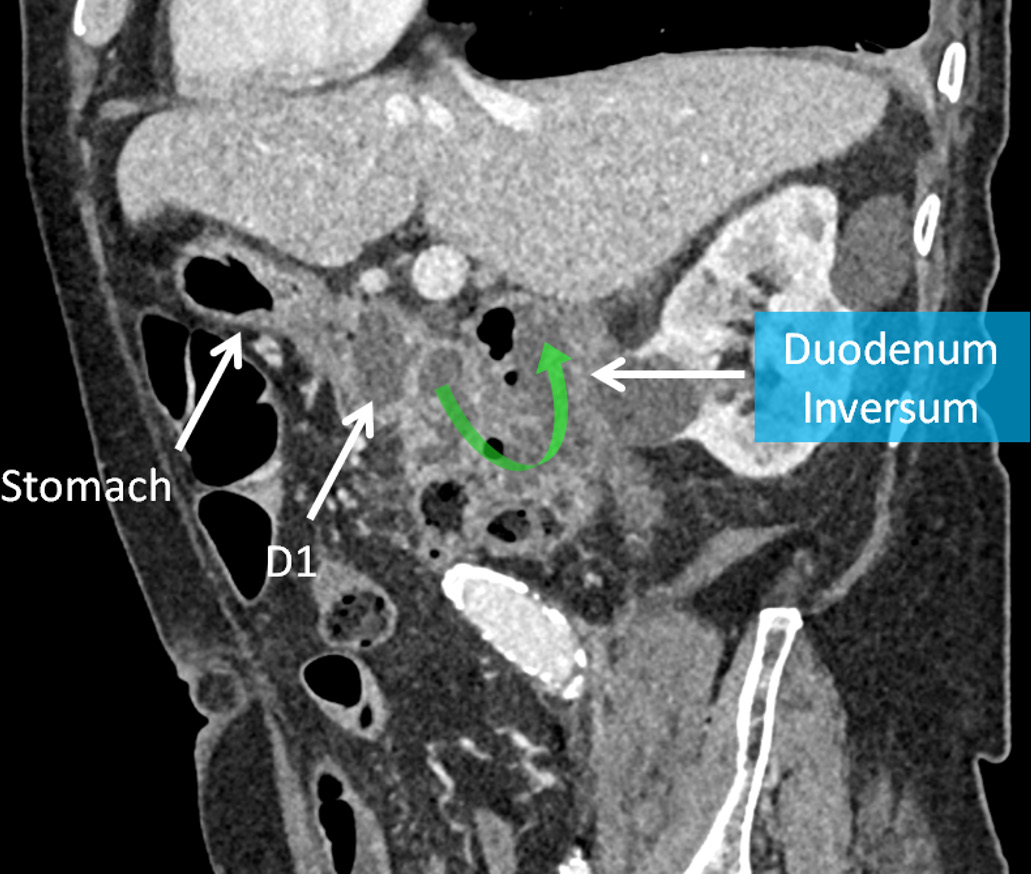
CT multiplanar reconstructed oblique sagittal image demonstrating duodenum inversum with the proximal duodenum travelling posteriorly and superiorly prior to crossing midline.

**Figure 3.**
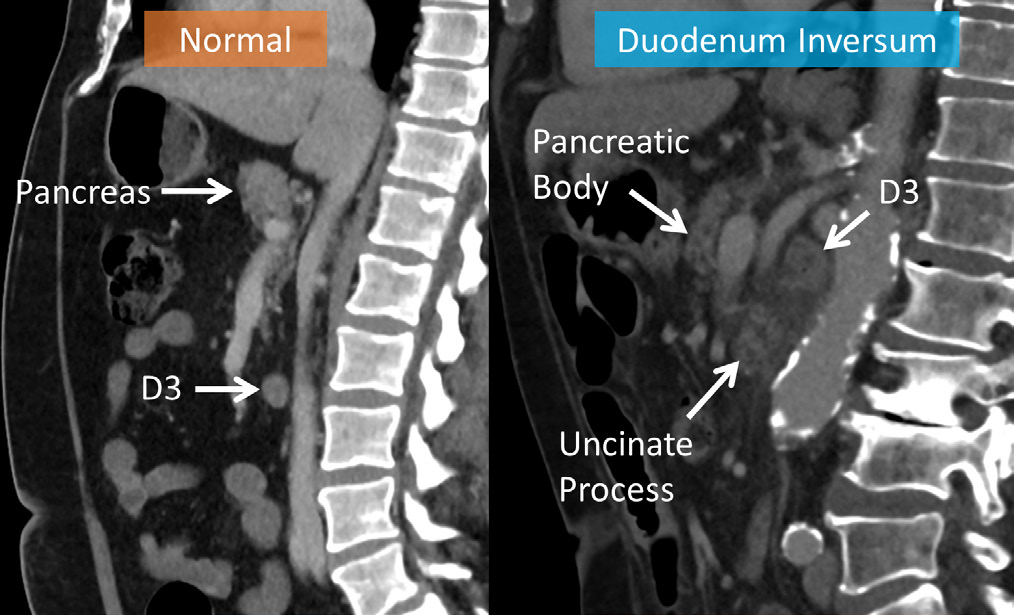
CT sagittal image demonstrating high positioning of the D3 segment of the duodenum in duodenum inversum compared to normal. Note the changes of duodenitis with abnormal wall thickening and surrounding fat stranding in the patient with duodenum inversum.

**Figure 4.**
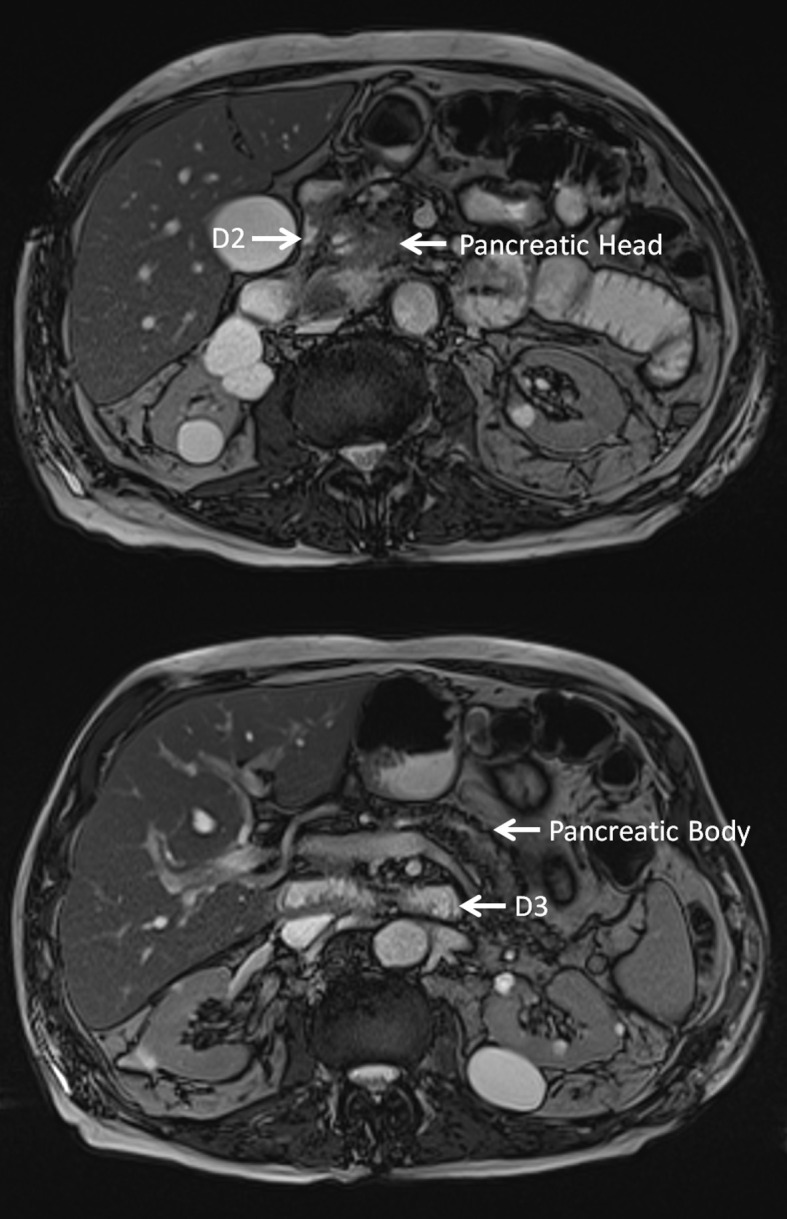
MRI TRUFI sequence axial images demonstrating the D3 segment of the duodenum at the same level of the pancreas.

Contrast-enhanced CT abdomen showed inflammatory stranding surrounding the D2 and D3 segments of the duodenum consistent with duodenitis. Close review of the duodenum showed an abnormal course with the proximal duodenum travelling posteriorly and superiorly prior to the D3 segment crossing midline in an abnormally high position, in keeping with duodenum inversum (Figures 1–4). Groove pancreatitis was ruled out given normal lipase level of 50 (0–60 U l^−1^) and no cystic thickening of the duodenal wall, fibrous tissue within the pancreaticoduodenal groove or common bile duct dilatation on imaging. Given the significant stranding in the region of the proximal duodenum and pancreatic head, further evaluation with MRI was performed to exclude a pancreatic head lesion and this was negative. The patient also underwent an emergent upper gastrointestinal endoscopy which confirmed duodenitis.

## Differential diagnosis

1. Intestinal malrotation: absence of a retromesenteric (retroperitoneal) D3 segment of the duodenum, abnormal SMA (smaller and more circular)–SMV relationship and large bowel predominantly on the left and small bowel predominantly on the right. These features are not present in duodenum inversum, as other than the abnormal course of the proximal duodenum, the large/ small bowel and SMA/ SMV’s anatomic relations remain preserved.
2. Redundant duodenum: elongated duodenal loop with normal position of the DJ flexure.^1^

## Management

The patient was given an intravenous bolus of 80 mg of pantoprazole whilst awaiting endoscopic and radiological investigations, followed by regular oral 40 mg pantoprazole twice a day for 3 months.

### Outcome and follow-up

The patient’s symptoms improved during admission. Repeat upper gastrointestinal endoscopic ultrasound 2 months later showed no significant sonographic signs of pathology in the pancreas and a benign-appearing intrinsic mild stenosis at the junction of the D1 and D2 segments of the duodenum.

## Discussion

Duodenum inversum is a congenital malformation where the proximal duodenum travels posterosuperiorly prior to crossing the midline in an abnormally high position.^2^ The incidence is rare (0.07%), with less than 20 cases reported since 1950.^3,4^ This anomaly appears to occur more commonly in males, with a 4:1 ratio reported.^3^ Aetiology is unknown, but it is hypothesised to be a result of the persistence of the dorsal mesentery resulting in a mobile duodenum.^3,5^ It can be complicated by duodenitis, acute pancreatitis, peptic ulcer disease and functional biliary obstruction.^3^ Associated conditions include malrotation, incomplete rotation, annular pancreas and pancreas divisum.^6^

Feldman and Morrison described four types of duodenum inversum: Type 1 is characterised by complete inversion of the duodenum with an absence of the duodenal curve; Type 2 is defined by the presence of the duodenal curve; Type 3 has a duodenal curve with marked redundancy of the duodenum; and Type 4 is defined as duodenum inversum associated with malrotation.^7^ However, the value of this, or any classification system, is limited by small numbers and a lack of correlation between specific anatomic variants and clinical presentation.^2^

Apart from CT and MRI, fluoroscopic upper gastrointestinal (GI) series with barium contrast have been used to illustrate multiple reported cases of duodenum inversum. Classic findings on upper GI series include: the return of contrast from the second part of the duodenum back into the first and then into the bulb; stasis in the duodenum; and rapid passage of contrast through the third part of the duodenum.^4,8^

Duodenum inversum is a rare condition that may be unfamiliar to many radiologists. However, the recognition of duodenum inversum is important especially because it usually responds well to conservative management and an accurate diagnosis will prevent unnecessary surgical investigations.

## Learning points

- Duodenum inversum is a rare congenital anomaly that is important to recognise and diagnose to avoid unnecessary surgical intervention.
- Diagnosis is established on fluoroscopy, CT or MRI based on characteristic abnormal course of the duodenum.
- It can be associated with duodenitis, acute pancreatitis, peptic ulcer disease and functional biliary obstruction.^3^
- Treatment of symptomatic duodenum inversum is usually medical therapy with proton pump inhibitors for patients with associated duodenitis or ulcers.^2,3,9^
